# Frost protection mechanism based on rubber airbag interlayered composite lining system for cold-region tunnels

**DOI:** 10.1038/s41598-024-57531-y

**Published:** 2024-03-21

**Authors:** Yuping Xu, Yang Liu, Wenge Qiu

**Affiliations:** Key Laboratory of Transportation Tunnel Engineering, Ministry of Education, School of Civil Engineering, Southwest Jiaotong University, Chengdu, 610031 China

**Keywords:** Cold-region tunnels, Rubber airbag, Composite lining system, Frost protection mechanism, Experimental study, Civil engineering, Engineering

## Abstract

To solve the problem of freezing damage in cold-region tunnels, this study proposed a rubber airbag interlayered composite lining system, and tested its performance of buffering, pressure adjustment, waterproofing, and heat preservation by simulating a low-temperature environment in an artificial freezing chamber. The experiment results show that the frost-heaving force exerted on the lining can always be lower than 1.69 kPa by constantly adjusting the airbag pressure, and the maximum frost-heaving force can reach 28.25 kPa without the airbag. In addition, the airbag also has good waterproof performance. Finally, the airbag can significantly improve the temperature field of the surrounding rock and reduce the freezing depth (6.75 cm < 17.25 cm). The insulation effect of the airbag is positively correlated with its thickness and negatively correlated with the thermal conductivity of the filling gas. The insulation effect of CO_2_ is better than that of air. The rubber airbag interlayered composite lining system provides a new scheme for freezing damage control in cold-region tunnels.

## Introduction

With the increasing demand for economic and social development, transportation infrastructure construction is expanding into high-altitude and cold regions. Alongside the construction and opening of numerous cold-region tunnels, various issues related to frost damage have emerged. These issues typically manifest as lining cracking, brittle spalling, leakage, ice accumulation, and freezing-induced expansion at the tunnel invert, which severely compromises the structural stability and traffic safety of the tunnels. Studies have indicated that groundwater, low-temperature environments, and frost-heaving force are the main causes of tunnel frost damage. Frost-heaving force is essentially the deformation pressure resulting from surrounding rock freezing, and the frost-heaving constraint state is a crucial factor in determining the magnitude of frost-heaving force.

The mainstream approach for frost damage prevention and control in cold-region tunnels involves the installation of a waterproof layer to prevent groundwater infiltration, the incorporation of insulation layers or heating systems to improve the temperature environment of the tunnel, and the utilization of flexible support systems to dissipate the energy from surrounding rock frost heaving and reduce the risk of damage to the lining structure.

In recent years, waterproofing, insulation, and flexible support technologies have been widely discussed and applied in cold-region tunnels^1^. Regarding frost protection, Liu et al.^2^ analyzed the fundamental factors causing frost damage in permafrost tunnels and the variation patterns of the freezing–thawing zone. They proposed a waterproofing and insulation structure form of “waterproofing board + insulation layer + waterproof protection layer” for permafrost tunnels of the Qinghai–Tibet Railway, which serves as a reference for similar engineering designs. Sun et al.^3^ studied the technology of tunnel waterproofing and drainage through a case study of Huashan Tunnel in Jilin Province, and results showed that multiple waterproofing measures (such as grouting concrete, smooth drainage pipes in a round, lengthways and transverse direction, waterproof layer, waterproof concrete lining and the center drainage pipe of buried deeply, etc.) must be integrated into water-control works in tunnels in the high cold region. The frost damage caused by seepage becomes more pronounced when tunnel construction is located in cold region environments. Cui et al.^4^ made a study of the nano-modification effects on the water multiscale transport behaviors of concrete in winter construction environments. Results indicated that the addition of nano-materials can markedly increase the complexity of the pore structure and decrease the porosity, thereby enhancing the impermeability of concrete. Lai et al.^5^ found that the effect of the thermal insulation door on heating the air in the tunnel is better than that of the anti-snow shelter, so the thermal insulation doors installed at both ends of the tunnel are more suitable to prevent the tunnel from being damaged by frost than the anti-snow shelters. Cui et al.^6^ proposed a new off-wall insulation liner for high-speed railway tunnels in cold regions. The off-wall insulation liner exhibits excellent insulation capacity, high construction efficiency, and excellent economic benefits. Ji et al.^7^ proposed several new technologies(such as the buried drainage water-stop, the ring drainage pipe with a strip of the insulation layer, the plan of embedding pipes in the concrete lining on both sides to form electric melting ice, and so on) to prevent frost damage of tunnels in cold regions. Wang et al.^8^ put forward a new composite thermal insulation lining for the railway operational tunnel in cold regions. Lai et al.^9^ conducted long-term performance tests on tunnel waterproofing layer materials and found that freeze–thaw cycles significantly degrade the material performance; however, the resistance of ethylene–vinyl acetate to low-temperature cracking was minimally affected by these cycles, indicating its waterproofing effectiveness. Huang et al.^10^ conducted experimental research on the failure mechanism of tunnel waterproofing boards, and the results indicated that the surrounding rock deformation pressure, shotcrete surface smoothness, and material properties were the main factors causing damage and failure of waterproofing materials during construction and operation. The methods for installing insulation layers include the external insulation layer method, intermediate insulation layer method, double insulation layer method, and off-wall insulation layer method^11^. Among them, the external insulation layer method was first applied in the Daban Mountain Highway Tunnel in Qinghai Province, China^12^, and the intermediate insulation layer method was successfully applied in the Kunlun Mountain Tunnel in the same province^13^, exhibiting excellent thermal insulation effect. Deng et al.^14^ investigated the thermal insulation principle of tunnel lining structures with an off-wall insulation layer, demonstrating its good thermal insulation and frost protection effects. The heating methods adopted for tunnel heating include direct heating of the secondary lining, heating of the internal air of the tunnel, and heating of water channels^15^. According to the concept of energy underground engineering, Xia et al.^16^ proposed the application of energy geotechnical engineering techniques to tunnel heating schemes, and Wu et al.^17^ proposed a construction joint lining and back-source heating method using warm air and electric heating strips for tunnel heating. Lv et al.^18^ suggested placing U-shaped electric heating strips between the waterproofing layer and the lining for tunnel heating, all of which were effective for frost prevention. Zhang et al.^19–21^ in their research on frost protection measures in the Linchang Tunnel on Boya Highway in Inner Mongolia, China, utilized a ground-source heat pump heating system and derived the analytical solution for the thermal conduction of circular tunnel composite media, successfully solving the frost protection problem in the tunnel. Gao et al.^22^ proposed an air curtain insulation and heating system for cold-region tunnels that effectively solves the problem of frequent opening and closing of cold protection doors, which affects vehicle traffic while providing insulation and frost protection. Regarding surrounding rock support, K. Kovári^23^ pointed out that forcibly suppressing rock deformation can lead to excessive deformation pressure. MH Mussa et al.^24^ assessed the tunnel damage mechanism under impact load. SM Anas et al.^25,26^ used FEM to study the mechanical response mechanism of high-performance reinforced concrete slabs under impact load. Sun et al.^27^ studied frost-heaving stress in saturated clay under different constraint conditions through experimental methods and found that greater axial constraints result in higher frost-heaving stress. Therefore, relying solely on increasing the strength or thickness of the lining is insufficient for enhancing the frost-heaving resistance, as it may lead to increased frost-heaving forces. G. Anagnostou and L. Cantieni^28^ proposed a support scheme that reduces the rock deformation pressure via the filling of a compressible layer behind rigid support structures. Zhang et al.^29^ suggested utilizing the excellent deformation properties of foam concrete to absorb rock frost-heaving energy, this method is highly applicable to frost-heaving forces in the range of 0.6–0.9 MPa, according to calculations.

The relevant study has indeed achieved a lot of valuable results, but there are still many shortcomings, such as single structure–function, repeated input of materials, high operation energy consumption, difficult management and maintenance, and cannot be dynamically adjusted according to the actual situation. Therefore, this study proposed a rubber airbag interlayered composite lining system to solve the problem of freezing damage in cold-region tunnels. Using an artificial freezing chamber to simulate the low-temperature environment to test the properties of buffering, pressure adjustment, waterproofing, and heat preservation, and compare with traditional rigid lining. The study results will provide a new idea for the prevention and control of freezing damage in cold-region tunnels and have important engineering application value.

## Experimental preparation and methods

**Figure 1:**
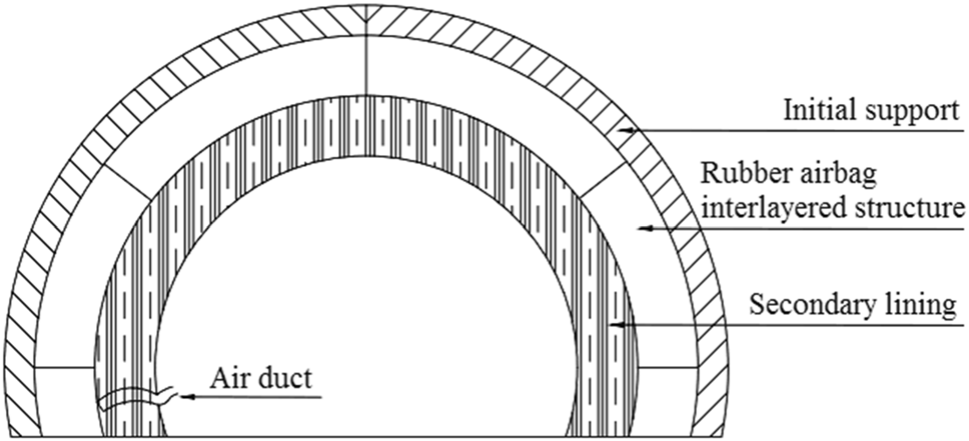
Cross section of the rubber airbag interlayered composite lining system.

**Figure 2:**
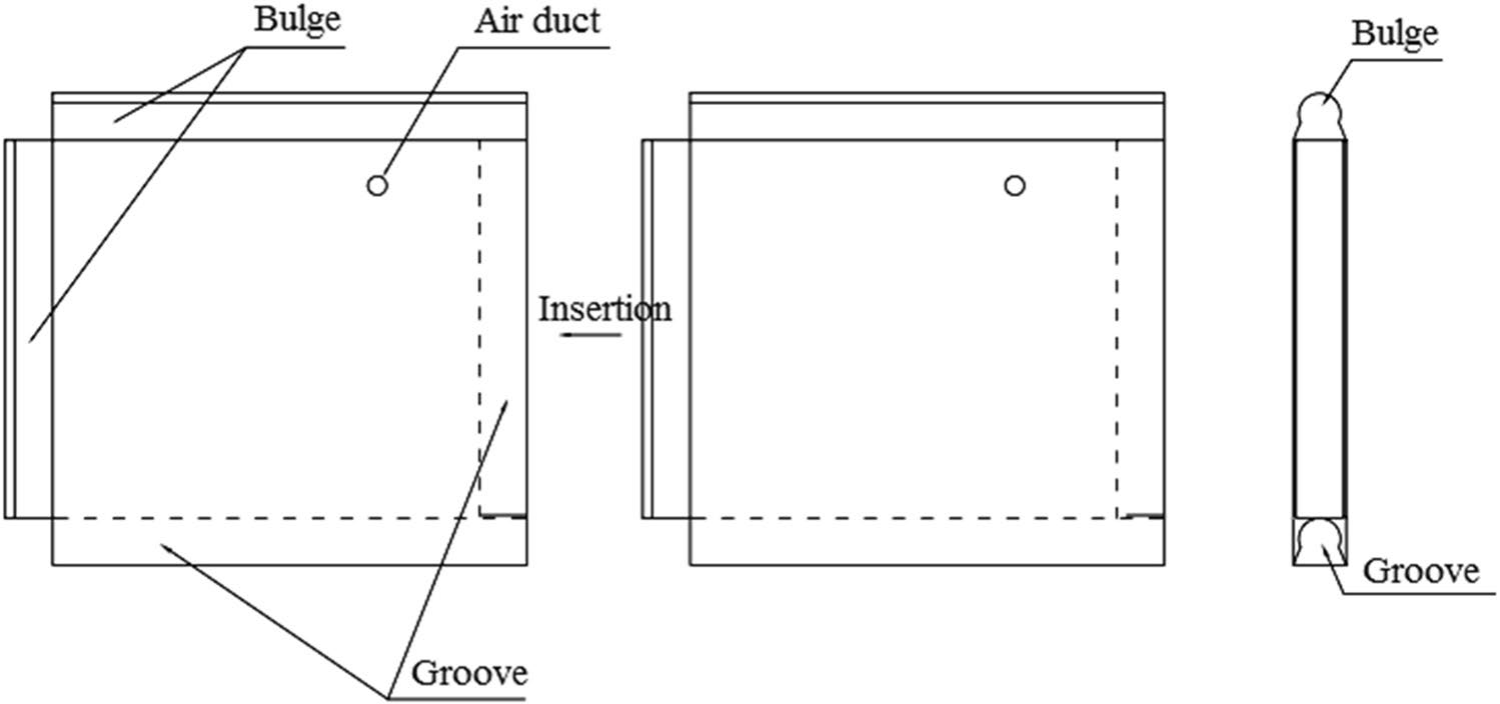
Splicing method of the rubber airbag interlayered composite lining system.

The rubber airbag interlayered composite lining system proposed in this paper for cold-region tunnels is a novel lining structure that incorporates a rubber airbag structural layer between the initial support and secondary lining, as shown in Fig. 1. The rubber airbag structural layer, which is composed of airtight hollow rubber bag units interconnected by their edge irregularities, uses water-expanding rubber in the irregular regions, as shown in Fig. 2. The airbags are connected to an air supply system, including inflation and deflation devices and monitoring instruments. They are filled with compressible gas with low thermal conductivity, and the extrusion forces from the internal pressure and water expansion pressure seal the joint gaps in a watertight manner. This structural layer provides pressure cushioning, monitoring, adjustment, waterproofing, and insulation functions, effectively addressing cold-region tunnel frost-related issues from multiple dimensions. It compensates for the shortcomings of traditional composite lining frost-heaving support and frost protection technologies and has considerable theoretical innovation and engineering application value.

### Experimental models

**Figure 3:**
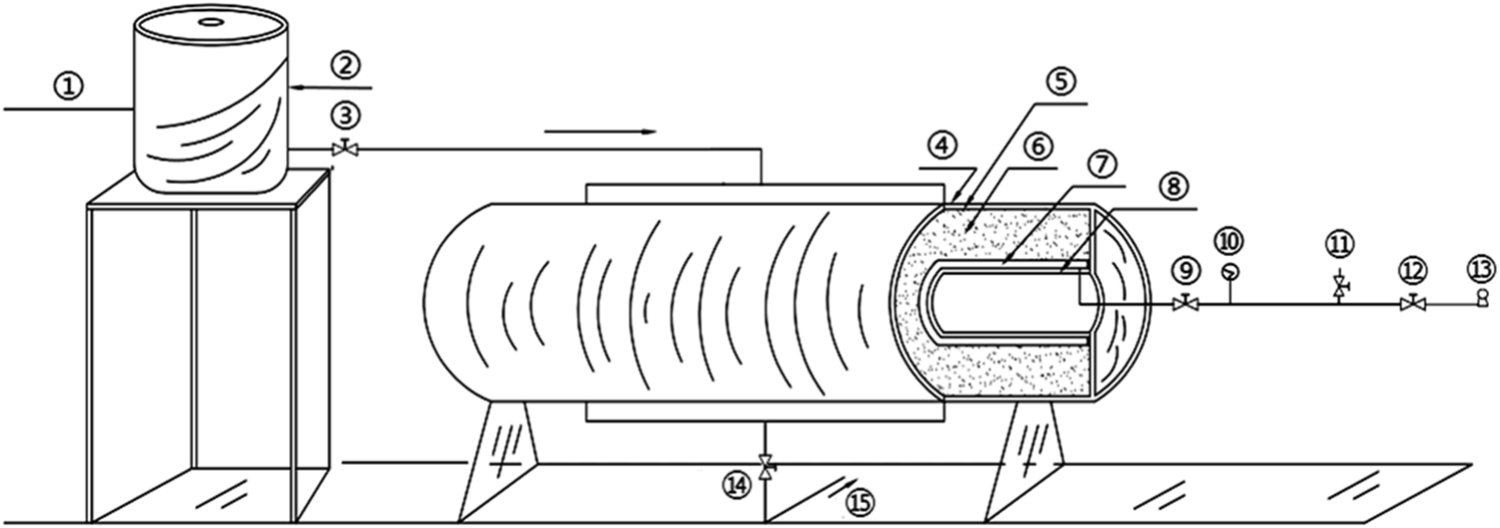
Experimental model.

**Figure 4:**
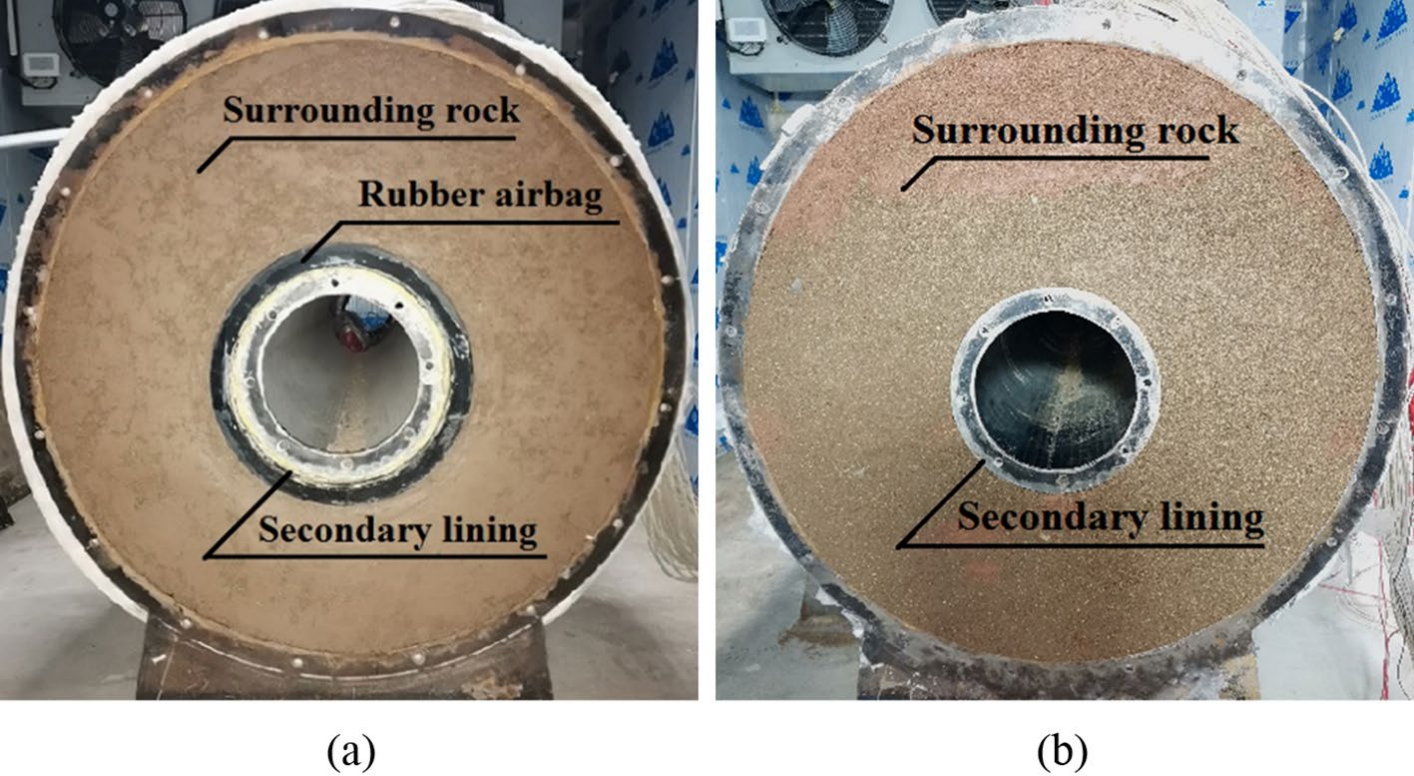
Cross sections of the experimental models. (**a**) Airbag model; (**b**) Airbag-less model.

Two experimental models were used: a circular tunnel with a rubber airbag interlayered structure (referred to as the “airbag model”) and a circular tunnel without a rubber airbag interlayered structure (referred to as the “airbag-less model”). Figures 3 and 4 present these models. The geometric similarity ratio of the models was 1:30, and the dimensions of the models were 300 mm (tunnel diameter) × 2400 mm (tunnel length).

Diagram description: ① Inlet pipe; ② Temperature-controlled water tank; ③ Inlet valve; ④ Heating strip and Insulation layer; ⑤ Surrounding rock confinement; ⑥ Surrounding rock; ⑦ Rubber airbag; ⑧ Secondary lining; ⑨ Air valve; ⑩ Pressure gauge; ⑪ Vent valve; ⑫ Inflation valve; ⑬ Air pump; ⑭ Drain valve; ⑮ Drain pipe.

### Experimental equipment and materials

#### Experimental equipment

**Figure 5:**
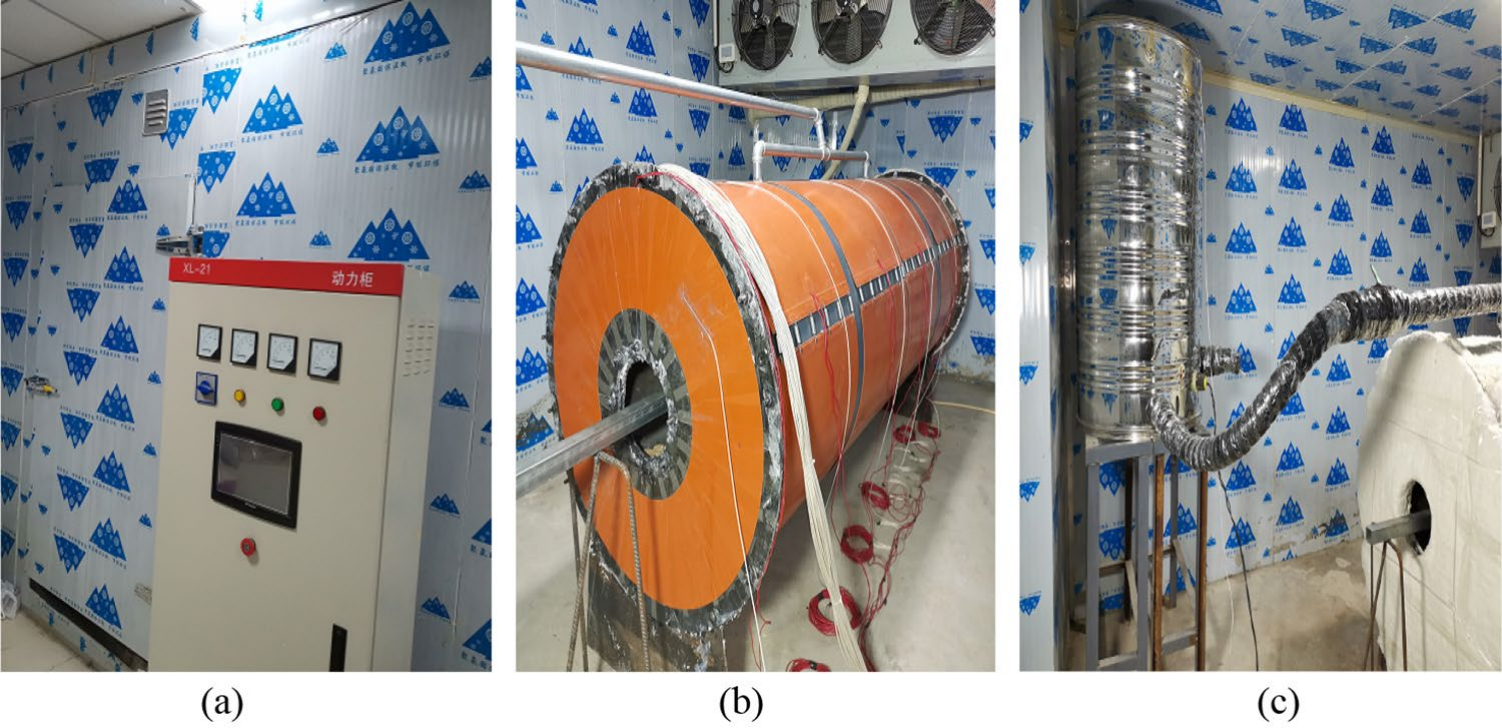
Experimental temperature control system. (**a**) Freezing chamber; (**b**) Electric heating strips; (**c**) Temperature-controlled water tank.

The temperature of the freezing chamber, the boundary temperature of the surrounding rock, and the water temperature were automatically controlled by a programmable logic controller control cabinet with an accuracy of ± 0.1 °C. The dimensions of the freezing chamber were 4.0 m × 4.0 m × 2.5 m, and the minimum temperature was – 35.0 °C. Electric heating strips were installed on the external surface of the model to regulate the boundary temperature of the surrounding rock, with a maximum temperature of 20.0 °C. A temperature-controlled water tank was used for water injection into the model’s surrounding rock, with a maximum temperature of 70.0 °C, as shown in Fig. 5.

Temperature measurement was performed using WZP-50AA waterproof thermal resistance temperature sensors with a resolution of 0.01 °C. Temperature data were acquired using an R70B data logger. Precise pressure gauges were used for measuring the pressure of the rubber airbag interlayered structure with a resolution of 0.30 kPa, and pressure data were visually collected. The frost-heaving force was measured using an HNY-1 pressure sensor with a resolution of 0.30 kPa. A TST3826F dynamic–static strain testing and analysis system was employed for frost-heaving force data acquisition.

#### Experimental materials

**Figure 6:**
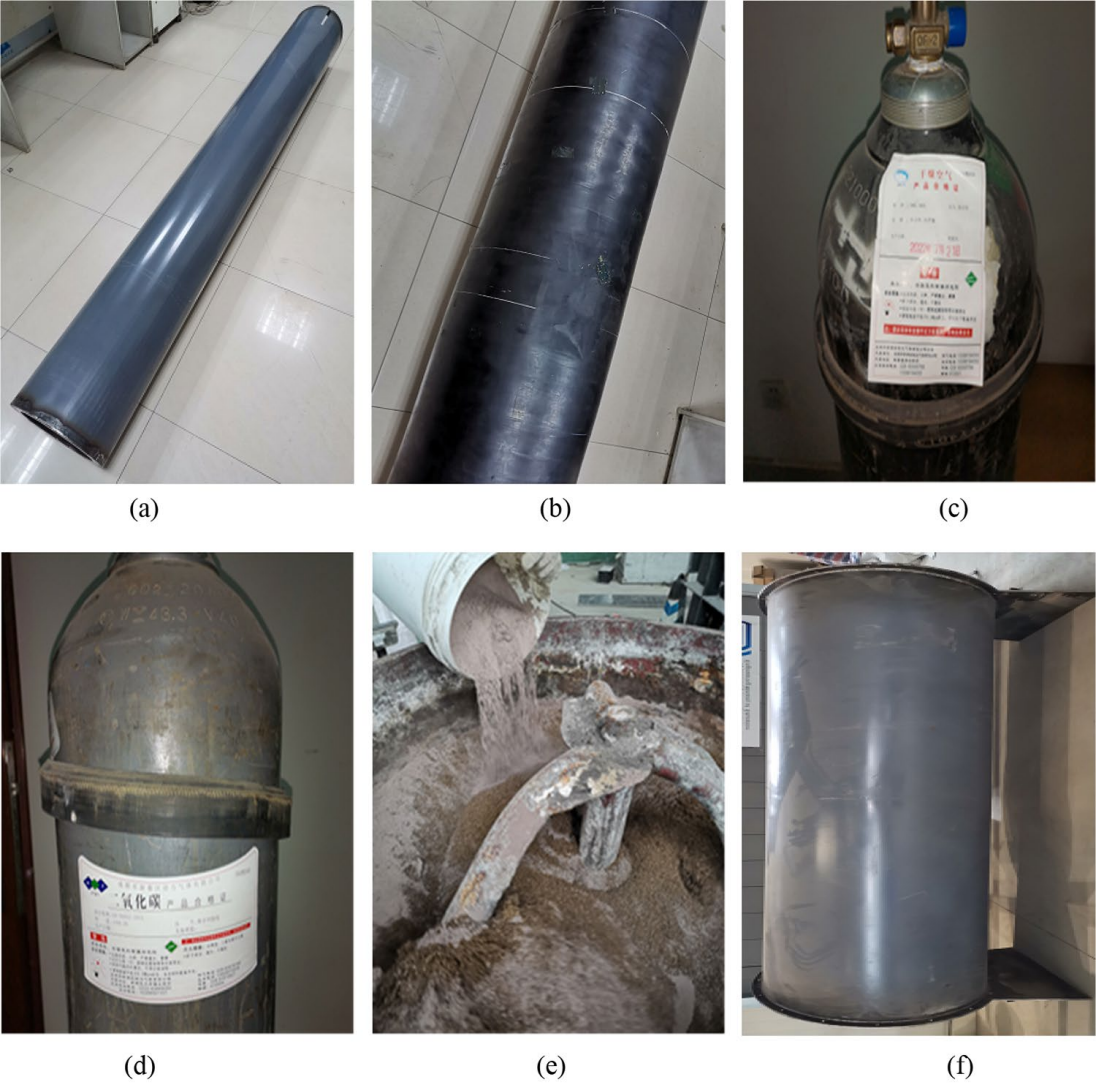
Experimental materials. (**a**) Inner steel cylinder; (**b**) Rubber airbag; (**c**) Air; (**d**) CO_2_; (**e**) Soil mixture; (**f**) External steel cylinder.

The secondary lining (referred to as the “inner steel cylinder” in the model) was made of low-carbon steel sheets with a thickness of 1.5 mm. It had a diameter of 300 mm and a length of 2400 mm, as shown in Fig. 6a. The rubber airbag was made of synthetic rubber, forming a sealed hollow cylindrical structure. Water-expanding rubber components were mixed into both ends of the airbag. The airbags were available in three specifications: 30, 40, and 50 mm. The thickness of the bag wall was 2 mm, and the thickness of the bag cavity was 26, 36, and 46 mm, respectively. The rubber airbag had an inner diameter of 306 mm and a length of 2400 mm, as shown in Fig. 6b. The inflation gas inside the rubber airbag interlayered structure was either air or CO_2_ gas, as shown in Fig. 6c and d, respectively. At 0 °C, the thermal conductivity (λ) of air was 0.024 W/(m·K), and for CO_2_ gas, it was 0.015 W/(m·K). The surrounding rock was prepared by mixing clayey soil and sand at a mass ratio of 15% to 85%. It exhibited a good water retention capacity and sensitivity to frost heaving, as shown in Fig. 6e. The outer steel cylinder (used for the confinement of the surrounding rock) was made of high-carbon steel sheets with a thickness of 5 mm. It had a diameter of 840 mm and a length of 2400 mm, as shown in Fig. 6f.

### Experimental methods and procedures

#### Buffering and pressure adjustment tests

The frost-heaving support function of the rubber airbag interlayered composite lining system primarily involves its reduction of the frost-heaving force of the surrounding rock. This is achieved through the pressure buffering and adjustment functions of the rubber airbag interlayered structure. The influence of the buffering function on the frost-heaving force is examined by adjusting the initial pressure of the airbag to alter the restraint state of the surrounding rock. During the freezing of the surrounding rock, the pressure was released promptly by deflating the airbag to study its effect on the frost-heaving force. The objective was to investigate the frost-heaving support mechanism and the effectiveness of the rubber airbag interlayered composite lining system. In contrast, the airbag-less model lacked the pressure adjustment function and did not reduce the frost-heaving force of the surrounding rock. This led to the accumulation of a significant amount of frost-heaving force. A comparison test was performed using the airbag-less model under the same conditions to highlight the functional advantages of the rubber airbag interlayered composite lining system. The test conditions designed according to the above ideas are presented in Table 1.

**Figure 7:**
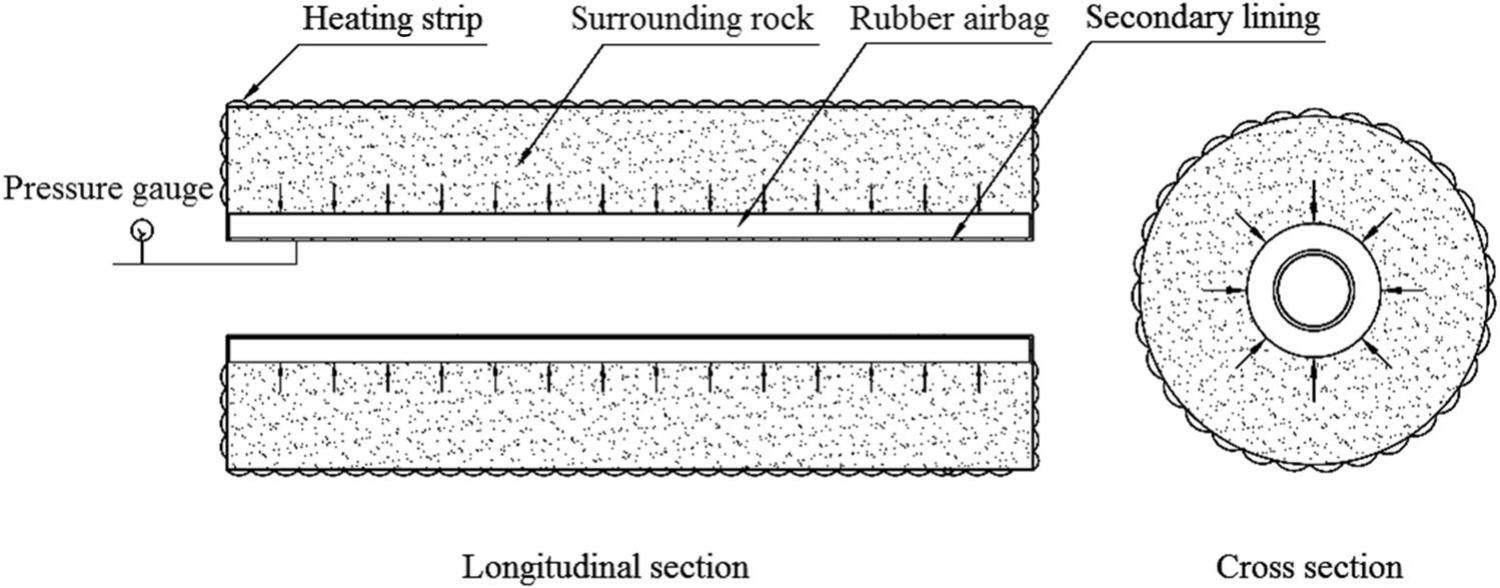
Monitoring method for the frost-heaving force in the airbag model’s surrounding rock.

**Figure 8:**
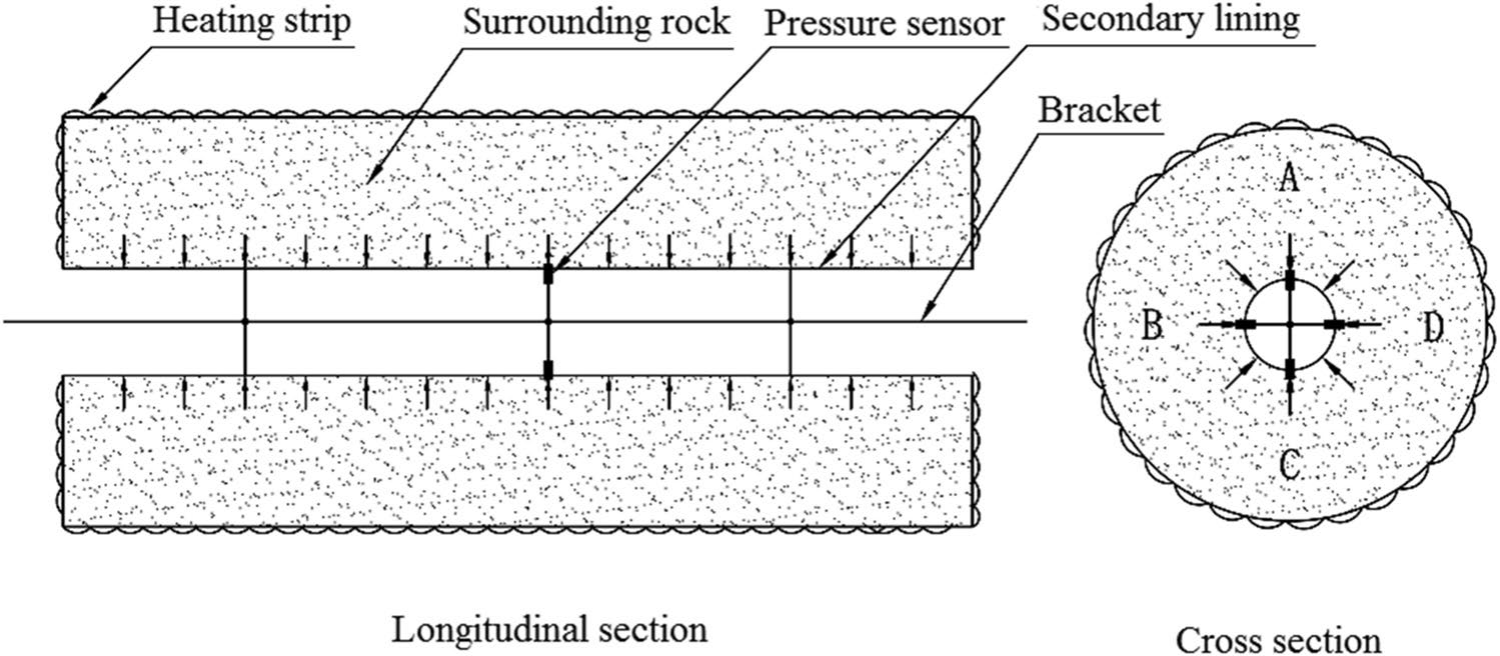
The layout of frost-heaving force measurement points in the airbag-less model’s surrounding rock.

1. Test conditions
2. Test sequence
3. A buffering test was conducted to examine the influence of the rubber airbag interlayered structure’s buffering function on the frost-heaving force.
4. A pressure adjustment test of the rubber airbag interlayered structure was conducted to examine its influence on the frost-heaving force. Additionally, the frost-heaving force was measured for the airbag-less model under the same conditions.
5. Experimental procedures
6. Setup and calibration of measurement devices: For the airbag model, the frost-heaving force measurement instrument was installed on the inflation line of the rubber airbag to measure the equilibrium value of the surrounding rock’s frost-heaving force, as shown in Fig. 7. For the airbag-less model, the frost-heaving force measurement instruments were arranged in four directions (A, B, C, D) on the cross-section of the model’s surrounding rock. The average value of the frost-heaving force was calculated, as shown in Fig. 8. The specific procedure involved inserting the pressure sensor through small holes in the secondary lining (inner steel cylinder) to contact the base point of the surrounding rock. The sensor was securely supported by a bracket, and waterproofing was applied at this location. Before the start of the experiment, calibration and initial reading recording of the instruments and acquisition devices were performed to ensure the accuracy of the test data.
7. The water temperature in the water tank was adjusted according to the boundary temperature values of the surrounding rock, and then water was injected into the model.
8. Once the surrounding rock reached the design moisture condition, with the heating element in the model not powered, the temperature in the freezing chamber was reduced to the boundary temperature of the surrounding rock. The model was maintained at this temperature for an adequate period.
9. When the temperature of the model’s surrounding rock (including the water) reached the boundary temperature value and the temperature distribution was uniform, the temperature of the heating element was set to the boundary temperature of the surrounding rock. Then, the power supply was connected to the heating element to maintain a constant boundary temperature of the surrounding rock throughout the test, using the heating element and insulation layer.
10. The temperature of the freezing chamber was set according to the design conditions, and the freezing test was initiated. In the airbag model test, the air in the freezing chamber exchanged heat with the secondary lining–the rubber airbag interlayered structural layer and the surrounding rock. In the airbag-less model test, the air in the freezing chamber exchanged heat with the secondary lining and the surrounding rock.
11. For the buffering function test on the frost-heaving force, the frost-heaving force was measured after the freezing of the surrounding rock reached equilibrium. For the pressure adjustment function test of the frost-heaving force, frost-heaving force data were collected at intervals of 3 h starting from the beginning of the freezing of the surrounding rock.
12. After the experiment was completed, the operation of the test system was stopped, and inspection and closure procedures were conducted.

**Table 1:** The buffering and pressure adjustment tests for the surrounding rock.

Category	Buffering test			Pressure adjustment test	
With/without airbag	With			With	Without
Airbag thickness (mm)	30			30	–
Internal gas	Air			Air	–
Test temperature	Air temperature – 20 °C, boundary temperature of surrounding rock 6 °C			Air temperature –30 °C, boundary temperature of surrounding rock 3 °C	
Surrounding rock water content	Saturated state				
Initial pressure (kPa)	18	13	8	13	–
Pressure adjustment time interval (h)	–			15	–

#### Waterproofing and frost protection tests

The frost protection effect of the rubber airbag interlayered composite lining system primarily manifests in the prevention of groundwater in the surrounding rock and thermal insulation of the rock. It relies on the waterproofing and thermal insulation functions of the rubber airbag interlayered structure. Tests were conducted to investigate the frost protection mechanism and effectiveness of the rubber airbag interlayered composite lining system, including tests of the waterproofing function, thermal insulation performance, and impact of thermal insulation on the temperature field of the surrounding rock. The airbag-less model without waterproofing or insulation is expected to result in severe frost damage. Thus, a comparison test was conducted using the airbag-less model without waterproofing or insulation to highlight the advantages of the rubber airbag interlayered composite lining system for frost protection. The experimental conditions designed according to these considerations are presented in Table 2.

**Figure 9:**
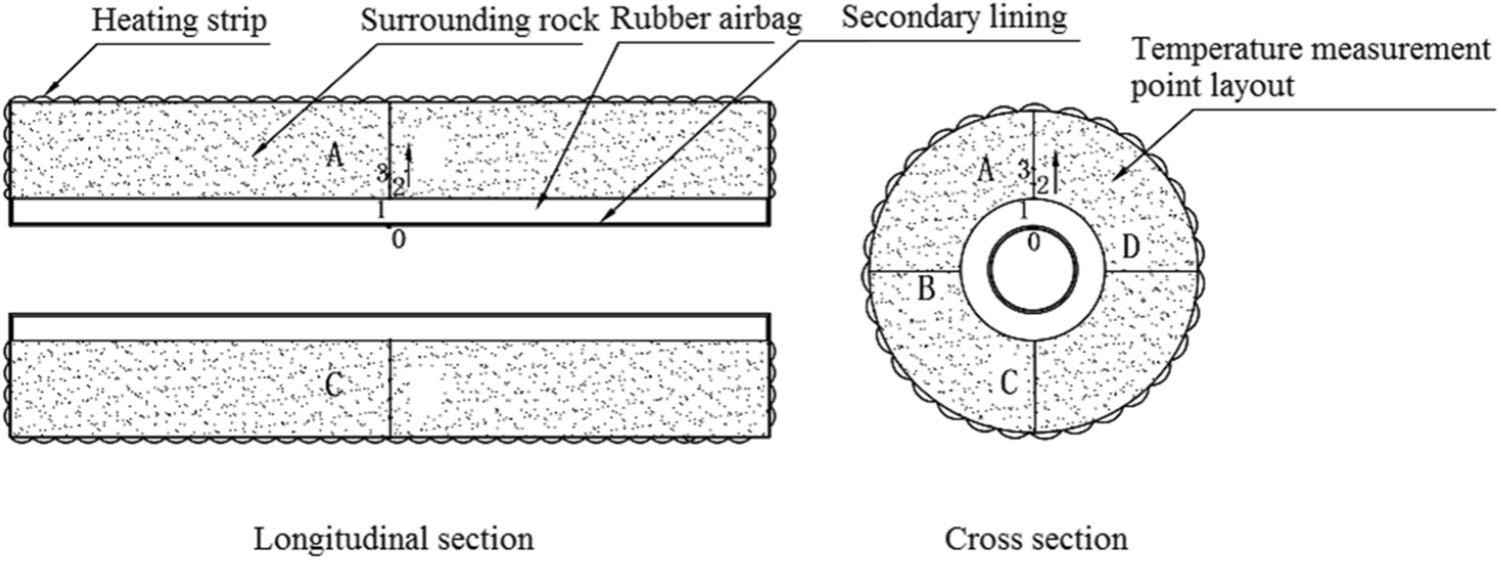
The layout of temperature measurement points in the surrounding rock of the airbag model.

**Figure 10:**
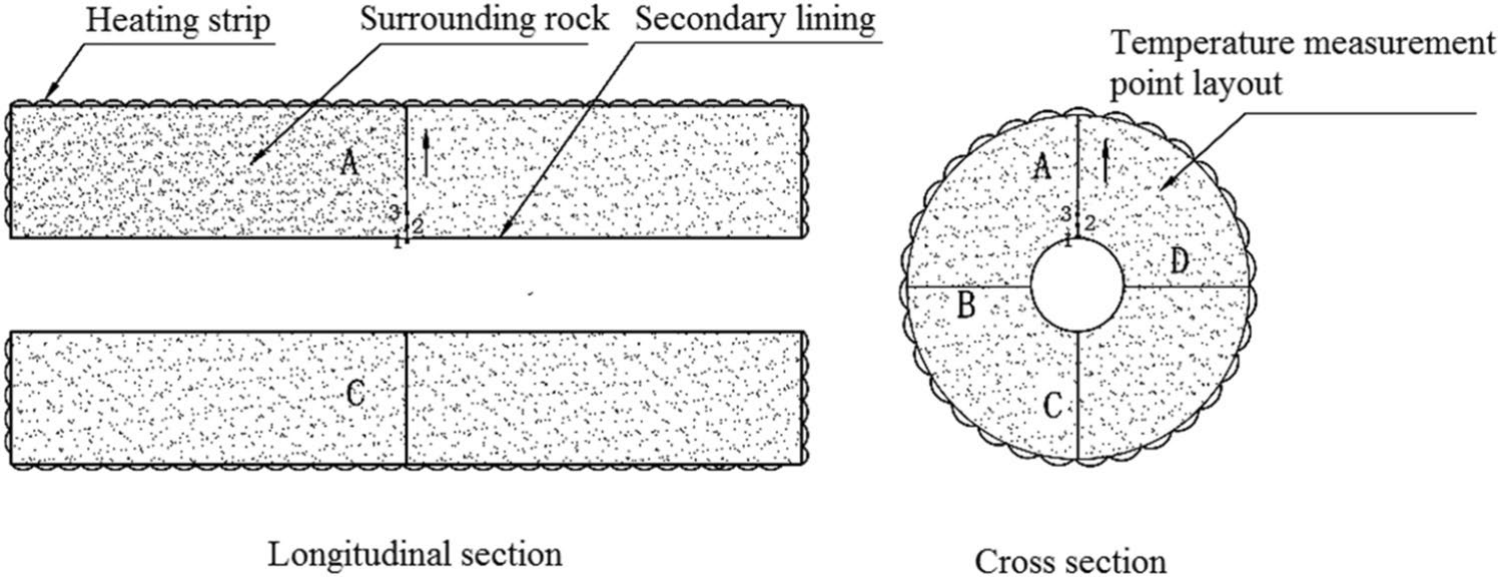
The layout of temperature measurement points in the surrounding rock of the airbag-less model.

1. Experimental conditions
2. Test sequence
3. A waterproofing test of the rubber airbag interlayered structural layer was conducted, and a seepage and freezing test of the airbag-less model was conducted under the same conditions.
4. A thermal insulation performance test of the rubber airbag interlayered structural layer was conducted.
5. The impact of the thermal insulation function of the rubber airbag interlayered structural layer on the temperature field of the surrounding rock was tested, and simultaneously, the temperature field of the surrounding rock in the airbag-less model was measured under the same conditions.
6. Experimental procedures
7. Installation and calibration of measurement devices: The layout of the temperature measurement elements in the surrounding rock was identical between the airbag and airbag-less models. The measurement points were set in four directions (A, B, C, D) on the cross-section of the model’s surrounding rock, with temperature measurement elements placed at intervals of 1.5 cm between measurement points. Additionally, temperature measurement elements were placed inside and on the outer side of the rubber airbag interlayered structural layer in the airbag model to measure the temperature difference between the inner and outer sides. Details are presented in Figs. 9 and 10. Before the start of the experiment, the temperature measurement elements and the acquisition system were calibrated to ensure the accuracy of the test data.
8. The water temperature in the water tank was adjusted according to the boundary temperature values of the surrounding rock, and then water was injected into the model.
9. Once the surrounding rock reached the design moisture condition, with the heating element in the model not powered, the temperature in the freezing chamber was reduced to the boundary temperature of the surrounding rock. The model was allowed to remain at this temperature for an adequate period.
10. When the temperature of the model’s surrounding rock (including the water) reached the boundary temperature value and the temperature distribution was uniform, the temperature of the heating element was set to the boundary temperature of the surrounding rock. Then, the power supply was connected to the heating element, and the heating element and insulation layer were used to keep the boundary temperature of the surrounding rock unchanged throughout the test.
11. The temperature of the freezing chamber was set according to the design conditions, and the freezing test was initiated. In the airbag model test, the air in the freezing chamber exchanged heat with the secondary lining–the rubber airbag interlayered structural layer and the surrounding rock. In the airbag-less model test, the air in the freezing chamber exchanged heat with the secondary lining and the surrounding rock.
12. During the waterproofing test, the phenomenon of water seepage and freezing in the model was examined. In the thermal insulation performance test and the test of the impact of thermal insulation on the temperature field of the surrounding rock, after the freezing of the surrounding rock reached equilibrium, data on the temperature difference between the inner and outer sides of the rubber airbag interlayered structural layer and the temperature field of the surrounding rock were collected at measurement point A, for example.
13. The experiment was concluded, the operation of the test system was stopped, and inspection and closure procedures were conducted.

**Table 2:** Frost protection test for the surrounding rock.

Category	Waterproofing test		Thermal insulation performance test	Thermal insulation test
With/without airbag	Without	With	With	With
Airbag thickness (mm)	–	30	30\40\50	30
Internal gas	–	Air	Air, CO_2_	Air
Test temperature	Temperature –15 °C, Surrounding rock boundary temperature 3 °C		Temperature – 20 °C, Surrounding rock boundary temperature 3 °C	Temperature – 25 °C, Surrounding rock boundary temperature 3 °C
Surrounding rock water content	Seepage state		Saturated state	
Initial pressure (kPa)	–	13	13	13

For the waterproofing test, a small number of fine holes were drilled in the inner steel cylinders of both the airbag and airbag-less models to simulate the secondary lining joints.

## Results and discussion

### Results of the buffering and pressure adjustment tests

For the airbag-less model, the frost-heaving force caused by the low-temperature frozen volume expansion of the saturated water-containing surrounding rock acts directly on the secondary lining. For the airbag model, the frost-heaving force caused by the low-temperature frozen volume expansion of the saturated water-containing surrounding rock first acts on the airbag, and then transfers to the secondary lining. The frost-heaving force data from the buffering and pressure adjustment tests are presented in Tables 3 and 4.

**Table 3:** Results of the frost-heaving buffering test on the surrounding rock.

Airbag initial pressure (kPa)	18	13	8
Frost-heaving force (kPa)	4.65	2.63	1.37

**Table 4:** Results of the frost-heaving force adjustment test on the surrounding rock.

Freezing time/h	Airbag model/kPa	Remarks	Airbag-less model/kPa
0	0.00	The full release of the frost-heaving force at 1.46 kPa	0.00
3	0.12		0.60
6	0.21		1.50
9	0.39		2.35
12	0.79		3.32
15	1.46		4.16
	0.00		
18	0.13	The full release of the frost-heaving force at 1.57 kPa	5.50
21	0.23		6.73
24	0.42		8.71
27	0.83		10.54
30	1.57		13.32
	0.00		
33	0.15	The full release of the frost-heaving force at 1.69 kPa	16.52
36	0.26		19.56
39	0.41		22.83
42	0.86		26.32
45	1.69		28.25
	0.00		28.25

The frost-heaving support effect of the rubber airbag interlayered composite lining system is mainly reflected in the positive effects of the cushioning function and pressure adjustment function of the rubber airbag interlayered structural layer on the frost-heaving force of the surrounding rock.

**Figure 11:**
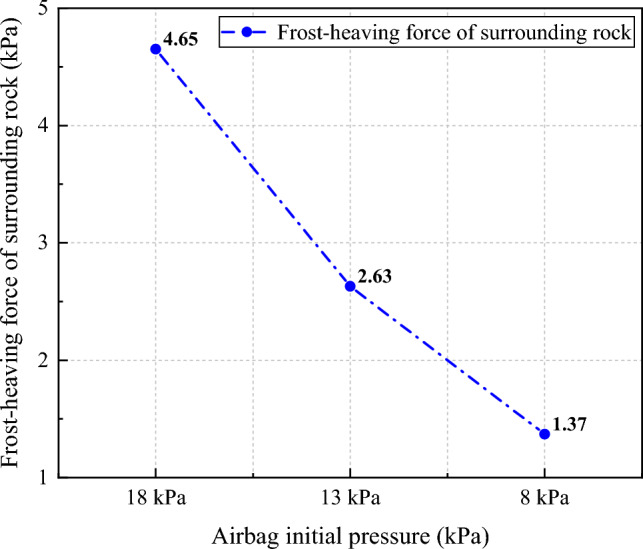
Relationship between the frost-heaving force of the surrounding rock and the initial pressure of the airbag.

The results of the buffering test indicated that after the freezing equilibrium of the surrounding rock, the airbag model with an initial pressure of 18 kPa generated a frost-heaving force of 4.65 kPa, the airbag model with an initial pressure of 13 kPa generated a frost-heaving force of 2.63 kPa, and the airbag model with an initial pressure of 8 kPa generated a frost-heaving force of 1.37 kPa. A lower initial pressure of the rubber airbag interlayered structural layer corresponded to a weaker frost-heaving force generated by the surrounding rock. The pressure cushioning function of the rubber airbag interlayered structural layer significantly affected the frost-heaving force of the surrounding rock, as shown in Fig. 11.

The underlying mechanism is as follows: when the water-containing surrounding rock undergoes frost-heaving deformation at low temperatures, it exerts pressure on the rubber airbag interlayered structural layer. Owing to the compressibility of the rubber material and the internal inflation gas, the rubber airbag interlayered structural layer relaxes the constraint on the frost heaving of the surrounding rock, changing the rigid constraint of the secondary lining on the frost heaving of the surrounding rock. Thus, the frost-heaving energy can cause the surrounding rock to undergo deformation and displacement toward the secondary lining. According to the principle of energy transformation through work done on an object, a lower initial pressure of the rubber airbag interlayered structural layer corresponds to a more relaxed frost-heaving constraint on the surrounding rock. The deformation and displacement of the surrounding rock caused by the frost-heaving energy will be larger, leading to more consumption of frost-heaving energy and effectively releasing the frost-heaving energy. This significantly weakens the frost-heaving force acting on the secondary lining and achieves the stability of the lining.

The results of the pressure adjustment test indicated that in the airbag model, during a freezing process lasting 45 h at an air temperature of – 30 °C, the frost-heaving force was released by three instances of pressure relief, totaling 4.72 kPa. Eventually, the secondary lining bore no frost-heaving force (0 kPa). In contrast, the airbag-less model accumulated a frost-heaving force of 28.25 kPa under the same conditions, with the entire force acting on the secondary lining. The pressure adjustment function of the rubber airbag interlayered structural layer significantly affected the frost-heaving force of the surrounding rock, and this model exhibited outstanding functional advantages over the airbag-less model.

**Figure 12:**
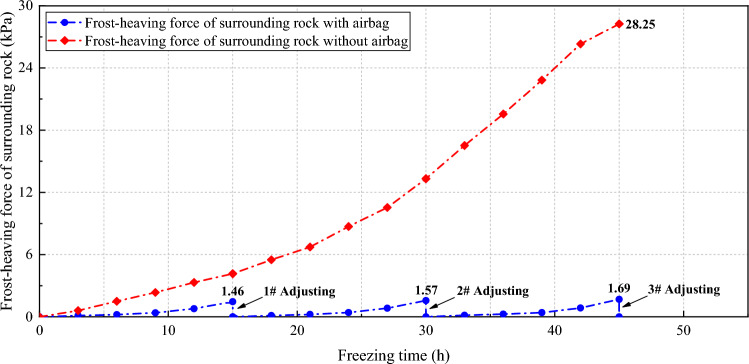
Trends of the frost-heaving force of the surrounding rock in the airbag and airbag-less models during the pressure adjustment test.

The underlying mechanism is explained as follows. Owing to the pressure adjustment function of the airbag model, it can actively intervene in the frost-heaving force during the freezing process through pressure adjustments of the rubber airbag interlayered structural layer. By conducting three instances of pressure relief, the frost-heaving force is consistently controlled within a certain range. The frost-heaving force curve exhibits a wave-like fluctuation, eventually decreasing sharply to zero, indicating a complete release of the frost-heaving force. This provides excellent protection to the secondary lining. In contrast, the airbag-less model lacks pressure adjustment capability, resulting in the accumulation of a significant amount of frost-heaving energy. The trend of the frost-heaving force curve remains consistently high compared with the airbag model, which is highly detrimental to the secondary lining. Figure 12 shows the variation trends of the frost-heaving force in the airbag and airbag-less models during the pressure adjustment test.

### Results of the waterproofing test

**Figure 13:**
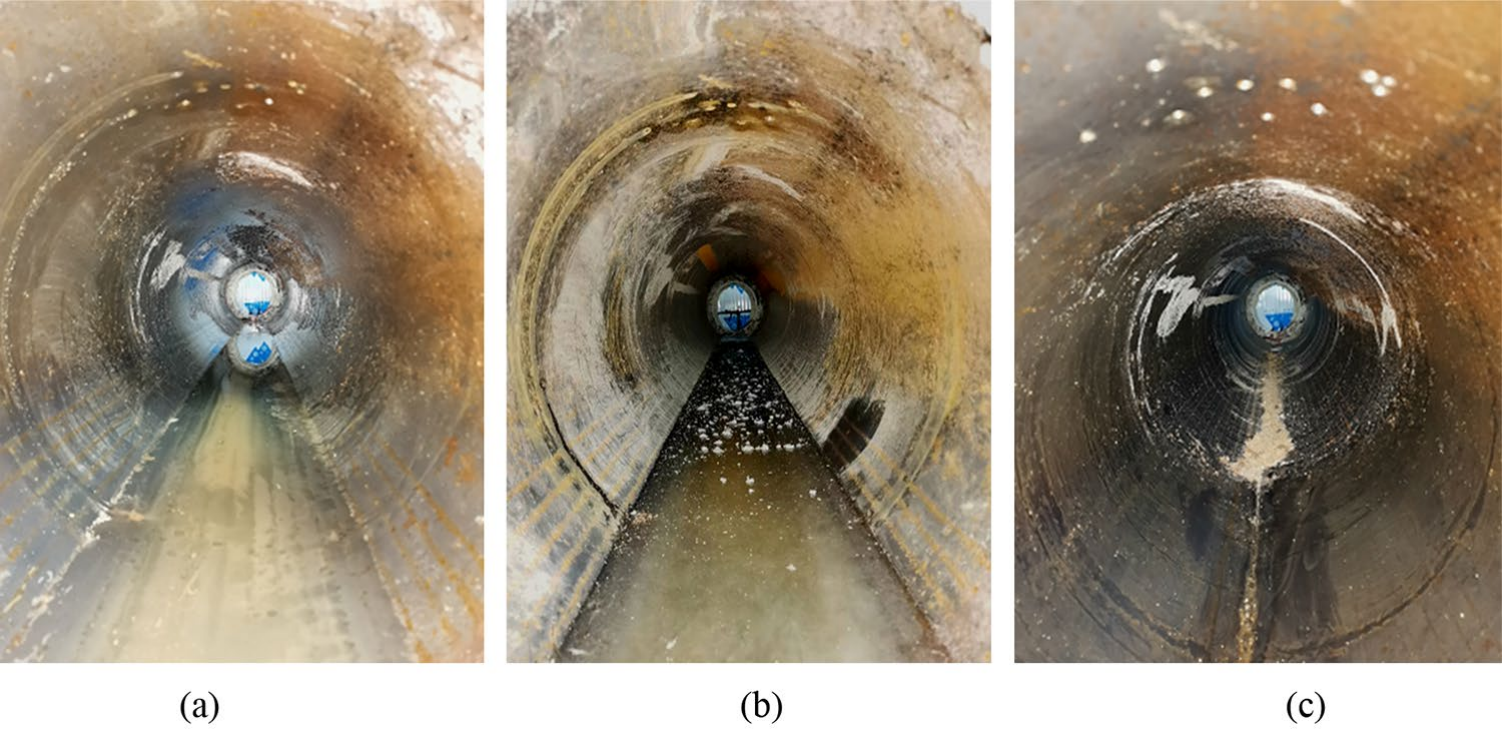
Comparison of waterproof performance between the airbag and airbag-less models. (**a**) Water seepage in the airbag-less model; (**b**) Ice formation in the airbag-less model; (**c**) No water seepage or ice formation in the airbag model.

The test results for the waterproofing of the airbag and airbag-less models are shown in Fig. 13.

In the test, the airbag-less model exhibited severe water seepage and ice formation. This is because no waterproof measures were taken. When water infiltrated the secondary lining (inner steel cylinder) through fine pores in the surrounding rock, it froze at low temperatures, accumulating as ice at the bottom of the tunnel, as shown in Fig. 13a and b.

In contrast, the airbag model exhibited no water seepage or ice formation, demonstrating a significant waterproof effect. This is because when water infiltrated the rubber airbag interlayered structural layer within the surrounding rock, the impermeability of the rubber material prevented water from flowing toward the secondary lining (inner steel cylinder). When water infiltrated the gaps at the ends of the model, the inflation pressure inside the rubber airbag structural layer and the expansion and compression of the rubber material upon contact with water sealed the gaps, preventing water from passing through. Therefore, no water seepage or ice formation occurred inside the tunnel, as shown in Fig. 13c.

### Results of the insulation test

The temperature differences between the inner and outer sides of the rubber airbag structural layer with different thicknesses and different internal gases are presented in Tables 5 and 6, respectively.

**Table 5:** Temperature differences between the inside and outside of the structural layer of rubber airbags with different thicknesses.

Thickness (mm)	30	40	50
Temperature difference (°C)	9.26	12.77	16.80

**Table 6:** Temperature differences between the inside and outside of the rubber airbag structural layer with different internal gases.

Internal gas	Air	CO_2_
Temperature difference (°C)	9.26	15.22

The test results indicated that under the same internal gas in the rubber airbag structural layer, the temperature difference between the inner and outer sides varied significantly with different thicknesses. The temperature difference was 9.26 °C for a thickness of 30 mm, 12.77 °C for a thickness of 40 mm, and 16.80 °C for a thickness of 50 mm. Similarly, under the same thickness of the rubber airbag structural layer, the temperature difference between the inner and outer sides differed significantly for different internal gases. The temperature difference was 9.26 °C for air and 15.22 °C for CO_2_.

**Figure 14:**
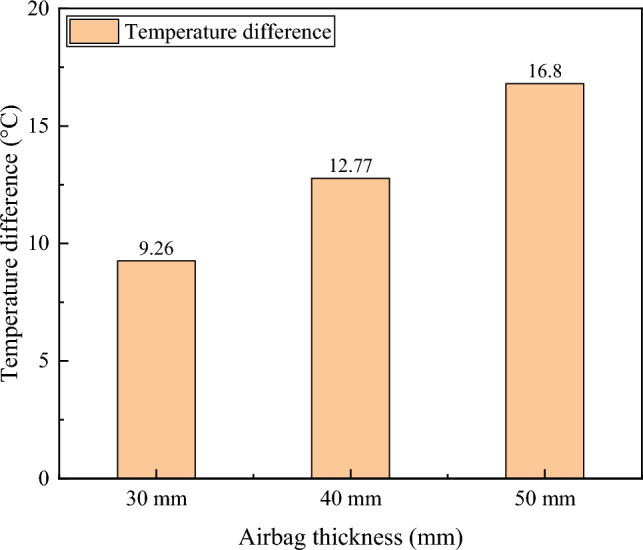
The thermal insulation performance of the rubber airbag structural layer with different thicknesses.

**Figure 15:**
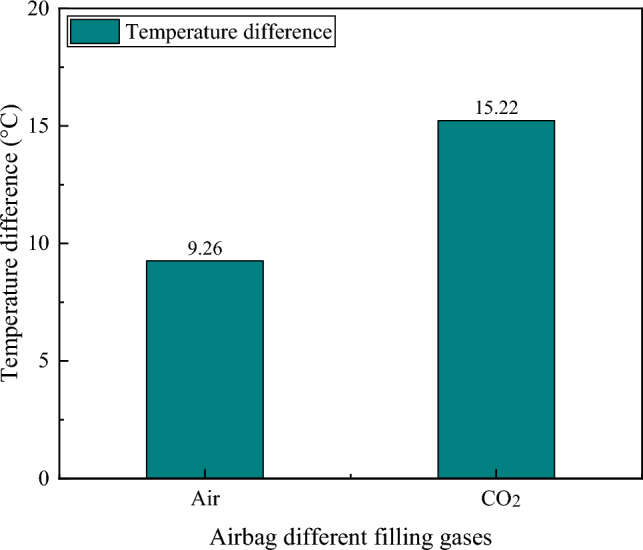
The thermal insulation performance of the rubber airbag structural layer with different internal gases.

The test results indicated that the rubber airbag structural layer had good thermal insulation performance. Additionally, a larger thickness and lower thermal conductivity of the internal gas corresponded to better insulation performance, as shown in Figs. 14 and 15. Therefore, in practical applications, the thickness and internal gas of the rubber airbag structural layer should be selected appropriately according to the temperature of the cold-region tunnel environment and the dimensions of the tunnel cross-section to ensure that the thermal insulation performance satisfies the requirements for insulation and frost protection in the tunnel.

### Results of the surrounding rock temperature field test

The experimental data for the surrounding rock temperature field are presented in Tables 7 and 8.

**Table 7:** Initial freezing time of the surrounding rock in the airbag and airbag-less models.

Model	Without	With
Initial freezing time (h)	16.2	52.0

The freezing of the surrounding rock is marked by the appearance of 0 °C at the base point on the outer surface of the secondary lining/rubber airbag structural layer. The initial freezing time refers to the time interval between the appearance of 0 °C at the base point and the start of the freezing test.

**Table 8:** Measurement results for the temperature field of the surrounding rock in the airbag and airbag-less models (direction A).

Measurement points	Depth of measurement (cm)	The measured temperature of the airbag-less model (°C)	The measured temperature of the airbag model (°C)
1	0	− 11.78	− 2.85
2	1.5	− 10.13	− 2.20
3	3.0	− 8.67	− 1.58
4	4.5	− 7.38	− 1.00
5	6.0	− 6.17	− 0.46
6	7.5	− 5.20	0.08
7	9.0	− 4.31	0.60
8	10.5	− 3.44	1.10
9	12.0	− 2.59	1.59
10	13.5	− 1.85	2.05
11	15.0	− 1.12	2.38
12	16.5	− 0.42	2.67
13	18.0	0.27	2.85
14	19.5	0.91	2.91
15	21.0	1.54	2.96
16	22.5	2.13	2.98
17	24.0	2.66	3.00

The frost protection effect of the rubber airbag interlayered composite lining system is primarily reflected in its positive effects on the initial freezing time, temperature field distribution, and freezing depth of the surrounding rock through its thermal insulation function. The test results indicated the following.

Under the same conditions, the time at which the surrounding rock started freezing differed significantly between the airbag-less and airbag models. In the airbag-less model, freezing occurred after 16.2 h, whereas in the airbag model, freezing occurred after 52.0 h. The surrounding rock in the airbag model froze later than that in the airbag-less model.

**Figure 16:**
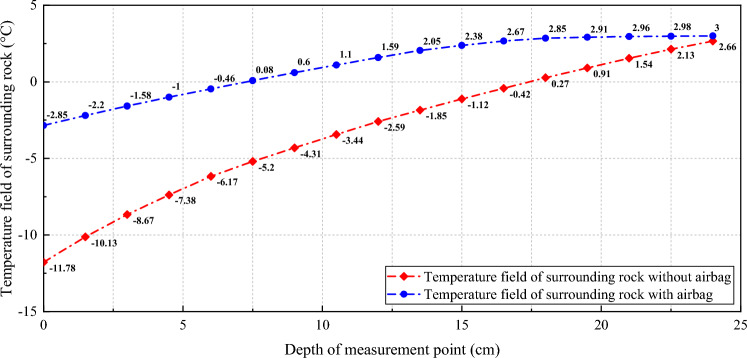
Temperature field distribution law of the surrounding rock in the airbag and airbag-less models.

After the freezing equilibrium of the surrounding rock was reached, the temperature at the measurement point 1 (base point) in the airbag-less model was – 11.78 °C, at point 5 was – 6.17 °C, at point 10 was – 1.85 °C, that at point 15 was 1.54 °C, and that at point 17 was 2.66 °C. In the airbag model, the temperature at the measurement point 1 was – 2.85 °C, at point 5 was – 0.46 °C, at point 10 was 2.05 °C, at point 15 was 2.96 °C, and at point 17 was 3.00 °C. Both models exhibited an increase in the temperature of the surrounding rock along the radial direction of the tunnel, but the temperatures at various measurement points were higher for the airbag model than for the airbag-less model, as shown in Fig. 16.

The position of the 0 °C line (freezing surface) after the freezing equilibrium of the surrounding rock was reached differed significantly between the two models. In the airbag model, it stopped between measurement points 5 and 6, whereas in the airbag-less model, it stopped between measurement points 12 and 13. This indicated that the maximum freezing depth of the surrounding rock in the airbag model was between 6.0 and 7.5 cm, which was significantly smaller than that (between 16.5 and 18.0 cm) in the airbag-less model.

This is because the rubber airbag structural layer in the airbag model, which is filled with air, had a low thermal conductivity and high thermal resistance. It formed a thermal insulation layer between the model’s surrounding rock and the secondary lining, which impeded heat transfer between the surrounding rock and the air inside the tunnel. This reduced the rate of temperature decline in the surrounding rock, delaying the onset of freezing. In addition, it inhibited the heat exchange between the surrounding rock and the air inside the tunnel, reducing the heat loss from the surrounding rock. This improvement in heat retention enhanced the distribution of the surrounding rock temperature field and reduced the depth of freezing in the surrounding rock.

Based on the present study results, the next step will be to study the frost protection performance of the rubber airbag interlayered composite lining system under different tunnel section types and surrounding rock porosity conditions.

## Conclusions

To address the frost-heaving problem of surrounding rock in cold-region tunnels, this paper proposed a rubber airbag interlayered composite lining system, whose buffering, pressure adjustment, waterproofing, and heat preservation properties were tested through laboratory experiments. The conclusions are as below.

1. The frost-heaving force of surrounding rock transferred to the secondary lining can be kept at a low level (≤ 1.69 kPa) by adjusting the airbag pressure several times during the freezing process, which is much lower than the 28.25 kPa of the airbag-less model.
2. Compared with the airbag-less model, the airbag model showed good waterproof performance without water seepage and icing.
3. The thermal insulation performance of the rubber airbag interlayered composite lining system improves with an increase in the airbag thickness and a reduction in the thermal conductivity (CO_2_ < Air) of the filling gas.
4. Compared with the airbag-less model, the airbag model has obvious advantages in delaying the initial freezing time (52 h > 16.2 h) of surrounding rock and reducing the freezing depth after freezing equilibrium (6.75 cm < 17.25 cm).
5. Suggesting to apply the rubber airbag interlayered composite lining system in the cold-region tunnels freezing damage control, and continue to improve its anti-freezing performance.

## Data Availability

The datasets used and/or analyzed during the current study are available from the corresponding author upon reasonable request.
